# Prognostic Value of Biomarkers in Children and Adolescents With Orthostatic Intolerance

**DOI:** 10.3389/fped.2021.752123

**Published:** 2021-11-23

**Authors:** Huijuan Yan, Shuo Wang, Hong Cai, Juan Zhang, Ping Liu, Yuwen Wang, Runmei Zou, Cheng Wang

**Affiliations:** ^1^Department of Pediatric Cardiovasology, Children's Medical Center, The Second Xiangya Hospital, Central South University, Changsha, China; ^2^Department of Neonatology, Xiangya Hospital, Central South University, Changsha, China

**Keywords:** biomarkers, prognosis, children, adolescents, orthostatic intolerance

## Abstract

Orthostatic intolerance (OI) refers to a series of symptoms that occur during upright standing, which can be relieved when returned to the supine position. OI is a common cause of syncope in children and adolescents. In recent years, more and more studies have been carried out to assess the prognosis of OI by using biomarkers, among which, flow-mediated vasodilation, left ventricular ejection fraction and fractional shortening, hemodynamic change during head-up tilt test, detection of 24-h urinary sodium excretion, body mass index, midregional pro-adrenomedullin, and erythrocytic H_2_S producing rate are relatively stable, inexpensive, and easy to obtain. With the help of biomarkers, individualized treatment can be carried out to improve the long-term prognosis of children and adolescents with OI. This article reviews the prognostic value of biomarkers in children and adolescents with OI.

## Introduction

Orthostatic intolerance (OI) is a series of symptoms during upright standing that can be relieved when returned to the supine position, such as lightheadedness, headache, fatigue, visual difficulties, pallor, palpitations, nausea, and sweating (1). OI is a clinical syndrome of autonomic regulation disorders. Head-up tilt test (HUTT) is an important method for diagnosing OI. OI is mainly divided into several hemodynamic types, including vasovagal syncope (VVS), postural tachycardia syndrome (POTS), orthostatic hypotension (OH), and orthostatic hypertension (OHT). VVS and POTS, the main forms of pediatric OI, are underlying causes of neurally mediated syncope (NMS), which is defined as syncope due to autonomic nerve dysfunction (2, 3). Hu et al. (4) reported that the incidence of syncope in children and adolescents aged 2~18 years in Changsha was 17.37%, with significant gender differences in different age groups. Bayram et al. (5) and Li et al. (6) reported that 30–50% of children experienced at least one episode of syncope until the adolescent period, most of whom were females and VVS accounted for 60–80% of all pediatric syncope. Acute OI, such as VVS, usually manifests as syncope, which is a transient loss of consciousness (TLOC) and body balance disorder due to transient cerebral ischemia, characterized by a rapid onset, short duration, and spontaneous recovery (7). Two main groups of TLOC are “TLOC due to head trauma” and “non-traumatic TLOC,” and the diagnosis of VVS should exclude other causes of non-traumatic TLOC such as epileptic seizures and psychogenic pseudosyncope (7, 8). Chronic OI is defined as OI that presents for at least 3 months, an example is POTS (9). The symptoms of POTS in adolescents usually appear in early puberty, after the age of 9 years old, and are more common in females than males (10, 11). Compared with VVS, syncope occurs less frequently in POTS, but most adolescent patients experience fatigue and some form of chronic pain (12). Its pathophysiology is heterogeneous, and the course may vary from patient to patient (13, 14), while comorbidity types and treatment measures can affect short- and long-term outcomes (15). Clinical symptoms of OI may fade or be relieved by the end of the process of the physical changes of puberty, or may accompany patients for a lifetime (16, 17), but it is not associated with significant mortality (18–20).

Abnormal Bezold–Jarish reflex, high level of catecholamine, and dysfunction of the autonomic nervous system, etc., may play important roles in the pathophysiology of VVS (6). Hypovolemia, peripheral vascular dysfunction, hyperadrenergic stimulation, and abnormality of the autonomic nervous function were thought to be involved in the pathogenesis of POTS (21). The current treatments for OI mainly include non-pharmacological therapy (health education, autonomic nervous function exercise, and increasing the intake of water and salt), pharmacological therapy (midodrine hydrochloride and metoprolol) and pacemaker therapy (3). The majority of patients respond to a combination of physical methods as well as pharmacotherapy (22), and can anticipate a full and complete recovery (23, 24). Although OI is a functional cardiovascular disease with self-limitation and favorable prognosis (25, 26), the occurrence of symptoms can seriously affect the physical and mental health, learning ability, and quality of life of children (27, 28), so it is of great necessity to find simple indicators to describe the prognosis of OI. Biomarkers can be used for qualitative or quantitative testing to reflect the changes in disease conditions and assess the efficacy. They provide an objective basis for guiding clinical judgment on the prognosis of VVS and POTS in children and adolescents, which is of great clinical value. Current biomarkers for evaluating the prognosis of OI mainly include flow-mediated vasodilation (FMD) (29), left ventricular ejection fraction (LVEF) and fractional shortening (LVFS) (30), 24-h urinary sodium excretion (31), body mass index (BMI) (32), mid-regional fragment of pro-adrenomedullin (MR-proADM) (33), erythrocytic hydrogen sulfide (H_2_S) producing rate (34), heart rate (HR), etc. Since there are few studies on biomarkers for other types of OI such as OH and OHT, we have not yet retrieved the relevant literature that meets the requirements. This article provides a review of the prognostic value of biomarkers, predictors of treatment efficacy, and recurrence, for OI, especially for VVS and POTS.

## Biomarkers in Prognostic Assessment of Pediatric Vasovagal Syncope

### Predictors of Therapeutic Efficacy in the Management of Pediatric Vasovagal Syncope

Multiple biomarkers have predictive value for the therapeutic efficacy of pediatric VVS. Wu et al. (35) and White et al. (36) found that abnormal regulation of vascular endothelium function may be involved in the occurrence of VVS. Color Doppler ultrasound detection of FMD changes in the brachial artery is a non-invasive examination that can reflect vascular endothelial function in children with VVS. Zhang et al. (37) found a significant increase in FMD in children with VVS compared with healthy children (11.93 ± 4.46 vs. 8.46 ± 2.18 %, *p* < 0.05). The enhancement of FMD of blood vessels during postural changes in children with VVS may lead to blood stasis in the lower extremities and abdomen, which leads to syncope through the Bezold–Jarish reflex. Zhang et al. (29) found that FMD in children with VVS after treatment of midodrine hydrochloride (MD) was significantly lower than that before treatment (11.07 ± 3.11 vs. 7.64 ± 1.81%, *p* < 0.001), and FMD in patients with good therapeutic efficacy was significantly higher than that with poor therapeutic efficacy (11.93 ± 2.83 vs. 7.80 ± 1.63%, *p* < 0.01). For a FMD of 8.85% as cutting value to predict efficacy of MD for treating VVS, the ROC curve showed that the area under the curve (AUC) was 0.895, the sensitivity and specificity of which were 90.0 and 80.0%. FMD could be a predictor of the efficacy of MD for treating children with VVS. The status of high catecholamine is one of the pathogenesis of VVS (38), and a certain dose of catecholamine such as dobutamine can increase LVEF and LVFS in humans with normal cardiac function (39, 40). Therefore, LVEF and LVFS may reflect the level of plasma catecholamine to an extent. Song et al. (30) followed up 30 children with VVS after metoprolol treatment, the LVEF and LVFS in the reactive group were significantly higher than those in the non-reactive group (LVEF: 72.8 ± 2.8 vs. 65.5 ± 4.6%, *p* = 0.001; LVFS: 41.1 ± 1.9 vs. 35.8 ± 3.6%, *p* = 0.002). To predict the efficacy of metoprolol intervention for 6 months, when the AUC was 0.906, with LVEF of 70.5% as a cutoff value, its sensitivity and specificity were 81.3 and 88.9%, respectively; when the AUC was 0.903, with LVFS of 37.5% as a cutoff value, its sensitivity and specificity were 93.8 and 66.7%, respectively. This study showed that children with VVS who had relatively high levels of LVEF and LVFS might achieve ideal therapeutic efficacy with β-blocker therapy. LVEF and LVFS, which are measured by echocardiography, are relatively stable, reliable, and safe. The increase in the level of catecholamine in the body can also be characterized by an excessive increase in HR. Zhang et al. (41) investigated the value of HR changes during HUTT and predictive value thereof in evaluating the efficacy of metoprolol therapy in children with VVS. It was found that the HR before positive response to HUTT was significantly higher in the effective treatment group than that of the ineffective treatment group (123 ± 15 vs. 96 ± 17 beats/min, *p* < 0.01), HR increment before positive response to HUTT showed significant difference among groups (42 ± 16 vs. 18 ± 13 beats/min, *p* < 0.01). Compared with that of the baseline value, if an increase of 30 beats/min in HR before positive response to HUTT was taken as a cutoff value, with respect to predicting the metoprolol efficacy in the treatment of VVS, the sensitivity was 81.0%, and the specificity was 80.0%. It may be more effective to choose β-blockers for those with a significant HR increase before positive response to HUTT. The QT interval dispersion (QTd) reflects the difference of electrical activity of cardiomyocytes in different parts of the ventricle, which is closely related to the autonomic nervous function in children. Meanwhile, autonomic dysfunction is one of the pathogenesis of VVS. Liu et al. (42) followed up 27 children with cardioinhibitory vasovagal syncope (VVS-CI). They found that QTd of the non-responsive group after intervention (non-drug intervention or oral rehydration salts) was longer than that of the responsive group (37 ± 4 vs. 29 ± 5 ms, *p* < 0.001). The AUC was 0.906. Taking QTd of 34.50 ms as the cutoff value, the sensitivity of predicting response to VVS-CI intervention was 90.0% and the specificity was 82.4% (Table 1). QTd of electrocardiogram has a good estimation value in the prognosis of VVS-CI in children and adolescents, but further research is needed to select specific therapy. In summary, LVEF has the largest AUC (0.906). Therefore, LVEF was chosen as a predictor of the efficacy of β-blocker therapy on VVS in children with priority.

**Table 1:** Predictors of therapeutic efficacy of pediatric VVS.

**References**	**Interventions**	**Biomarkers**	**Cutoff values**	**AUC**	**Sensitivity (%)**	**Specificity (%)**
Zhang et al. (29)	MD	FMD	>8.85%	0.895	90.0	80.0
Song et al. (30)	Metoprolol	LVEF	≥70.5%	0.906	81.3	88.9
		LVFS	≥37.5%	0.903	93.8	66.7
Zhang et al. (41)	Metoprolol	Increment of HR before positive response in HUTT	30 beats/min	-	81.0	80.0

*MD, Midodrine hydrochloride; FMD, Flow-mediated vasodilation; LVEF, Left ventricular ejection fraction; LVFS, Left ventricular short axis shortening; HR, Heart rate; HUTT, Head-up tilt test*.

### Risk Factors of Recurrence of Pediatric Vasovagal Syncope

Some biological indicators are valuable for the recurrence prediction of VVS. Hemoglobin concentration (HGB) can be used to estimate blood volume in the clinic. Kabul et al. (43) reported a close correlation between platelet count (PLT) and autonomic nerve. Song et al. (44) reported the blood routine parameters of 63 children with VVS and found that baseline HGB (*HR* = 1.055, 95% *CI:* 1.007–1.105), mean corpuscular hemoglobin (MCH) (*HR* = 0.612, 95% *CI:* 0.423–0.884), and PLT (*HR* = 1.015, 95% *CI:* 1.006–1.024) might be the influencing factors of the syncopal recurrence of VVS in children. The risk of future syncope events increased by 5.5 and 1.5% for each additional unit of HGB and PLT, and decreased by 38.8% for each additional unit of MCH. Ye and Ma (45) also reported the blood routine results in 82 children with VVS, and found that HGB, PLT, and MCH were higher in the recurrence group than those in the non-recurrence group (HGB: 135.91 ± 16.33 vs. 117.22 ± 15.74 g/L, *p* < 0.05; PLT: 259.95 ± 47.32 × 10^9^/L vs. 228.75 ± 55.33 × 10^9^/L, *p* < 0.05; MCH: 29.71 ± 3.52 vs. 22.10 ± 2.11 pg, *p* < 0.05). Increasing HGB, PLT, and MCH might be the risk factors of recurrence in children with VVS. Children in both studies were treated with basic treatment (including predisposing causes avoiding, standing training, autonomic nervous function exercise, and oral rehydration salts). Both studies demonstrated the relationship between HGB, PLT, and syncope recurrence, but the contrary results of MCH. As the sample size of the study is small, a multi-center large sample study is needed to increase the conviction and reliability of the results.

In recent years, the research on the indicators for predicting the recurrence of VVS has being continuously updated. Chronotropic competence refers to the function that the HR increases appropriately with the increase in the metabolic needs of the body under the action of various physiological and pathological factors (46). Zhang et al. (47) reported that the chronotropic competence was an important indicator of cardiac autonomic nervous function in children with VVS. They analyzed 28 children with VVS, of which four children with cardioinhibitory type had chronotropic incompetence (CI), while the incidence of CI in children with vasodepressor type was only 33.3%. VVS children with CI responded poorly to treatment (including health education, oral rehydration salt, metoprolol, or midodrine), and the recurrence rate of syncope was significantly higher than that of children without CI (52.9 vs. 10.0%, *p* < 0.05). This study suggests that CI may be a significant predictor for poor prognosis in children with VVS.

## Biomarkers in Prognostic Assessment of Pediatric Postural Tachycardia Syndrome

### Predictors of Non-pharmacological Therapy in Postural Tachycardia Syndrome

Physical therapy and sleep-promoting therapy are important parts of non-pharmacological therapy in children with POTS. Lu et al. (48) explored whether electrocardiography (ECG) variables could be used to predict responses to physical treatment in children with POTS. The results showed that 40 children with POTS had prolonged baseline QTd and HR-corrected QTd (QTcd) compared with healthy children, and a longer baseline QTcd for responders to physical treatment (69.2 ± 31.2 vs. 43.5 ± 25.9 ms, *p* < 0.05). When the AUC was 0.730, using 43 ms as a cutoff of QTcd, yielded a sensitivity of 90.0% and a specificity of 60.0%. Physical treatment is a safe and inexpensive approach and frequently used in the clinic, so QTcd has great clinical practical value. Circulating catecholamine excess is considered as one of the pathogenesis of POTS. The levels of the catecholamines have been found to correlate with cortisol levels (49). Follenius et al. (50) found that insufficient sleep or sleep disruption is associated with significant increases in plasma cortisol levels. Salivary cortisol concentrations have been used to predict the efficacy of sleep-promoting treatment in children with POTS since salivary cortisol levels reflect serum cortisol levels (51). Lin et al. (52) found that cortisol concentrations in children with POTS (40 cases) were significantly higher at all time points than those in the control group (*p* < 0.05 for all) and significantly higher in responders than in non-responders (4.83 ± 0.73 vs. 4.05 ± 0.79 ng/ml, *p* = 0.003). With the AUC of 0.758, salivary cortisol >4.1 ng/ml at awakening yielded 83.3% sensitivity and 68.7% specificity in predicting therapeutic efficacy of sleep-promoting treatment in POTS (Table 2). Salivary cortisol determination helps to prevent and manage sleep problems, which is of great significance to promote the physical and mental health of children with POTS. Therefore, QTcd and salivary cortisol can be used as predictors of non-drug treatment in POTS children.

**Table 2:** Predictors of therapeutic efficacy of pediatric POTS.

**References**	**Interventions**	**Biomarkers**	**Cutoff values**	**AUC**	**Sensitivity (%)**	**Specificity (%)**
Lu et al. (48)	Physical treatment	QTcd	≥43 ms	0.730	90.0	60.0
Lin et al. (52)	Promoting sleep	Salivary cortisol	>4.1 ng/ml	0.758	83.3	68.7
Zhang et al. (31)	ORS	24-h urinary sodium	<124 mmol/24h	0.879	76.9	93.0
Li et al. (32)	ORS	BMI	≤18.02 kg/m^2^	0.923	92.0	82.8
Lu et al. (53)	ORS	MCHC	>347.5 g/L	0.730	68.8	63.2
Lin et al. (54)	ORS	HRD between orthostatic and supine position HRmax in upright 10 min	≥41 beats/min ≥123 beats/min	0.780 0.690	84.0	56.0
Li et al. (55)	ORS	BRS	>17.01 ms/mmHg	0.855	85.7	87.5
Zhang et al. (33)	MD	MR-proADM	>61.5 pg/ml	0.879	100.0	71.6
Yang et al. (34)	MD	Erythrocytic H_2_S producing rate	≥27.1 nmol/min/10^8^ RBC	0.813	78.9	77.8
Liao et al. (56)	MD	FMD	≥9.85%	0.803	74.4	80.0
Zhang et al. (57)	Metoprolol	Norepinephrine	>3.59 pg/ml	0.785	76.9	91.7
Lin et al. (58)	Metoprolol	CNP	>32.55 pg/ml	0.821	95.8	70.0
Wang et al. (59)	Metoprolol	TR index SDNN index	TR ≤ 33.7 SDNN ≤ 79.0 ms	0.807 0.820	85.3	81.8
Wang et al. (60)	Metoprolol	HR5	≥110 beats/min	0.794	82.5	69.2
		HR10	≥112 beats/min	0.802	84.6	69.7
		HRD5	≥34 beats/min	0.905	85.3	89.5
		HRD10	≥37 beats/min	0.901	97.6	64.9

*ORS, Oral rehydration salts; MD, Midodrine hydrochloride; HR, Heart rate; QTcd, HR-corrected QT interval dispersion; BMI, Body mass index; MCHC, Mean corpuscular hemoglobin concentration; HRD, HR difference; HRmax, Maximum HR; BRS, Baroreflex sensitivity; MR-proADM, Midregional fragment of pro-adrenomedullin; Erythrocytic H_2_S, Erythrocytic hydrogen sulfide; FMD, Flow-mediated vasodilation; CNP, C-type natriuretic peptide; TR index, Triangular index; SDNN index, Standard deviation index of all sinus intervals; HR5, Instantaneous HR of HUTT at 5 min; HR10, Instantaneous HR of HUTT at 10 min; HRD5, Difference between instantaneous HR at HUTT 5 min and the baseline HR; HRD10, Difference between instantaneous HR at HUTT 10 min and the baseline HR*.

Hypovolemia has been reported to be associated with the onset of POTS (21). The sodium content of the body determines the volume of extracellular fluid, including plasma. Taking oral rehydration salts (ORS) is an effective way to increase the intake of water and salt, and multiple biological indicators have predictive value for the efficacy of ORS. Zhang et al. (31) explored whether 24-h urinary sodium excretion served as an indicator of the efficacy of ORS in children with POTS (30 cases). The results showed that 24-h urine sodium excretion of patients with POTS was lower than controls (117.09 ± 58.63 vs. 193.88 ± 91.12 mmol/24 h, *p* = 0.022). Symptom severity was negatively correlated with 24-h urinary sodium excretion (*r* = −0.754; *p* < 0.001). The AUC was 0.879. Taking the 24-h urine sodium concentration of 124 mmol/24 h as the cutoff value, the sensitivity and specificity of predicting the efficacy of POTS in children were 76.9 and 93.0%. The 24-h urine sodium excretion is a useful indicator because it can identify salt-deficient individuals and predict which ones will benefit most from increased salt intake. In addition, Li et al. (32) found that BMI in the POTS group (54 cases) was significantly lower than that in the control group (18.22 ± 3.23 vs. 20.62 ± 3.05 kg/m^2^, *p* < 0.01), and the BMI in responders to ORS was significantly lower than that of non-responders (16.32 ± 2.28 vs. 20.43 ± 2.74 kg/m^2^, *p* < 0.01). When the BMI was 18.02 kg/m^2^, the AUC was 0.923, and it had high sensitivity (92.0%) and high specificity (82.8%) for predicting the efficacy of ORS treatment for POTS (32). A study by Stewart et al. suggested that BMI was associated with blood volume (61). BMI is a stable and inexpensive predictor and can be measured readily in the outpatient setting. Lu et al. (53) reported that in 35 children with POTS, ORS as an intervention, the baseline mean corpuscular hemoglobin concentration (MCHC) values of responders was higher than that of non-responders (351.1 ± 9.0 vs. 341.5 ± 12.2 g/L, *p* < 0.05). The AUC was 0.73. The use of a cutoff value for MCHC of 347.5 g/L yielded a sensitivity of 68.8% and a specificity of 63.2% in predicting the effect of ORS for treating POTS. A study by Lin et al. showed that low red blood cell volume played an important role in POTS (62), which was associated with hypovolemic state. The MCHC may reflect the characteristics of the red blood cells and, thus, predict the effectiveness of ORS therapy. Lin et al. (54) reported the change in HR during the HUTT of 54 children with POTS, which showed that compared with the non-responding group, the HR change during HUTT was greater in the responding group before treatment (46 ± 10 vs. 37 ± 9 beats/min, *p* = 0.001), and the upright maximum HR (HRmax) in 10 min was also higher in the responding group (122 ± 12 vs. 113 ± 10 beats/min, *p* = 0.010). ORS for children with POTS would be predicted to be effective when the HR difference (HRD) between orthostatic and supine position was 41 beats/min and the HRmax in upright for 10 min was 123 beats/min before treatment, its sensitivity was 84.0% and specificity was 56.0% (54) (Table 2). HR changes in HUTT may help to quickly identify children with POTS who may benefit from ORS treatment clinically. Baroreflex sensitivity (BRS) plays an important role in the instantaneous regulation of blood pressure, which is related to autonomic function. Convertino and Baumgartner (63) found that increased BRS might be associated with low blood volume. Li et al. (55) found that children with POTS (45 cases) had a significantly higher BRS than that of healthy children (18.76 ± 9.96 vs. 10 ± 5.42 ms/mmHg, *p* < 0.01), and the baseline BRS was significantly higher in the treatment (ORS) effective group than that in the ineffective treatment group (24.7 ± 9.9 vs. 13.5 ± 6.6 ms/mmHg, *p* < 0.01). The AUC was 0.855. A cutoff value of BRS of 17.01 ms/mmHg yielded the predictive sensitivity of 85.7% and specificity of 87.5%. Detection of BRS could well predict the disease outcome of POTS, and it was convenient, inexpensive, and non-invasive in the prediction. In summary, 24-h urine sodium excretion, BMI, MCHC, HR and HRD, and BRS can all be used as predictors of efficacy. BMI has the largest AUC (0.923), so it was recommended as a predictor of the efficacy of ORS treatment for POTS children with hypovolemia with priority.

### Predictors of Pharmacological Therapy in Postural Tachycardia Syndrome

The MR-proADM, erythrocytic H_2_S producing rate, and FMD can help to predict the efficacy of MD on POTS (33, 34, 56). The MR-proADM is relatively stable and can reflect levels of adrenomedullin (ADM), which is related to vasodilation (64, 65). Peripheral vascular dysfunction is an important pathophysiological mechanism of POTS (21). Zhang et al. (33) found that plasma levels of MR-proADM in children with POTS (57 cases) were significantly higher than that in the control group [75.0 (62.5–96.0) vs. 58.5 (50.3–69.0) pg/ml, *p* < 0.01], and was higher in the effective group of MD treatment than that in the ineffective group [76.0 (66.0–91.0) vs. 59.0 (54.0–65.5) pg/ml, *p* < 0.01]. The AUC was 0.879, and taking 61.5 pg/ml of MR-proADM as the cutoff value, the sensitivity and specificity of predicting the efficacy of MD in the treatment of POTS were 100.0 and 71.6%, respectively. Therefore, the plasma level of MR-proADM can be taken as one of the reference indicators in choosing medication for children with POTS. H_2_S is a new vasodilating gasotransmitter (66), and endogenous H_2_S was primarily released from erythrocytes. Erythrocytic H_2_S-producing rate may play a role in abnormal vasodilation in children with POTS. Yang et al. (34) explored the role of erythrocytic H_2_S-producing rate in predicting the therapeutic efficacy of MD in children with POTS (28 cases). H_2_S production from erythrocytes was significantly higher in the POTS group than that in the control group (*p* < 0.01), and it was also significantly higher in responders to MD than non-responders (39.2 ± 17.5 vs. 23.3 ± 12.5 nmol/min/10^8^ RBC, *p* < 0.05). The AUC was 0.813. Using erythrocytic H_2_S producing rate of 27.1 nmol/min/10^8^ RBC as a cutoff value, the sensitivity and specificity for predicting efficacy were 78.9 and 77.8%, respectively. As a biomarker, erythrocytic H_2_S-producing rate is relatively stable, inexpensive, and simple to test. FMD and abnormal endothelial function may also play important roles in the development of POTS (67). Liao et al. (56) found that FMD values in children with POTS (108 cases) were significantly higher than those in controls (11 ± 3 vs. 6 ± 2%, *p* < 0.001), and that FMD values of MD responders were significantly higher than those in MD non-responders (11 ± 3 vs. 8 ± 2%, *p* < 0.05). The AUC was 0.803, and FMD of 9.85% had a high sensitivity (74.4%) and specificity (80.0%) for a 3-month therapy (Table 2). In general, MR-proADM has the largest AUC (0.879). It is suggested that MR-proADM should be chosen as a predictor of the efficacy of MD treatment for POTS children with vascular dysfunction with priority.

Metoprolol is a commonly used drug for POTS treatment in children, and recent studies have found that a variety of biomarkers can be used to predict the efficacy of metoprolol. They are of great importance for the individual therapy of POTS in hyperadrenergic children and adolescents. Increases in orthostatic plasma norepinephrine are the core of the biochemical changes of hyperadrenergic children with POTS (21). Zhang et al. (57) reported that the symptom severity in children with POTS (25 cases) was positively correlated with their orthostatic plasma norepinephrine level (*r* = 0.599; *p* < 0.001), and orthostatic plasma norepinephrine level in the response group to metoprolol was significantly higher than that in the non-response group (5.10 ± 2.69 vs. 2.93 ± 1.79 pg/ml, *p* = 0.028). The AUC was 0.785. Once orthostatic plasma norepinephrine level was ≥3.59 pg/ml, it predicted the efficacy of metoprolol on POTS with a sensitivity of 76.9% and specificity of 91.7%. In addition, Takekoshi et al. (68) and Springer et al. (69) separately found that plasma C-type natriuretic peptide (CNP) played a role in increasing the secretion of plasma catecholamine and accelerating the HR. The increased plasma level of catecholamine was suggested to be involved in the pathogenesis of POTS. Lin et al. (58) reported significantly higher plasma CNP levels in children with POTS (34 cases) than in healthy children (51.9 ± 31.4 vs. 25.1 ± 19.1 pg/ml, *p* < 0.001). They also found that plasma CNP in responders to metoprolol was significantly higher than that in non-responders (59.1 ± 33.5 vs. 34.8 ± 16.7 pg/ml, *p* = 0.037) before treatment. The AUC was 0.821. When the plasma CNP was >32.55 pg/ml, the sensitivity and specificity for predicting the efficacy of metoprolol were 95.8 and 70.0%, respectively. As a biomarker, plasma CNP cannot only predict the efficacy but also reflect the severity of the pathophysiology of children with POTS. Heart rate variability (HRV) is an important reference indicator of autonomic regulation and is also used in the efficacy prediction of metoprolol in children with POTS. Wang et al. (59) found that baseline triangular (TR) index and standard deviation index of all sinus intervals (SDNN index) were significantly lower in responders than in non-responders to metoprolol (TR: 27.3 ± 6.10 vs. 35.7 ± 7.2, *p* < 0.01; SDNN: 63.2 ± 12.8 vs. 84.5 ± 18.3 ms, *p* < 0.01) in 45 children with POTS. The AUC for TR index and SDNN index was 0.807 and 0.820, respectively. Combined baseline TR index ≤ 33.7 and SDNN index ≤ 79.0 ms as cutoff values, the sensitivity and specificity to predict efficacy of metoprolol were 85.3 and 81.8%, respectively. HRV indicators may be non-invasive and easy-to-use predictors. Wang et al. (60) found that HR and HRD during HUTT could predict the efficacy of metoprolol in children and adolescents with POTS. The results showed that HR5, HR10 (instantaneous HR of HUTT at 5 and 10 min, respectively), HRD5 and HRD10 (the difference between instantaneous HR at HUTT 5 and 10 min, and the baseline HR, respectively) were significantly higher in the group with POTS than those in the control group (*p* < 0.01). The AUC at HR5, HR10, HRD5, and HRD10 was 0.794, 0.802, 0.905, and 0.901, respectively. They found when HR5, HR10, HRD5, HRD10 ≥110, 112, 34, 37 beats/ min, respectively, the sensitivity and specificity to predict response to metoprolol were 82.5 and 69.2%, 84.6 and 69.7%, 85.3 and 89.5%, 97.6 and 64.9%, respectively (Table 2). The indicator is relatively simple and easy to obtain, but it is susceptible to changes in mood. Therefore, the HUTT procedures should be strictly followed to ensure the accuracy of the data collection. In summary, orthostatic plasma norepinephrine, plasma CNP, TR index and SDNN index, and HR and HRD can all be used as predictors of efficacy. HRD5 has the largest AUC (0.905), therefore it is recommended that HRD5 should be selected as a predictor of the efficacy of metoprolol treatment for hyperadrenergic children with POTS with priority.

Certainly, the research on the indicators that predict the therapeutic efficacy in the management of pediatric POTS is also constantly being updated. Wang et al. (70) reported changes in rate-pressure product (RPP) in children with POTS (53 cases). The results showed that when RPP at HUTT 5 min (RPP5) was 11,548.5 bpm·mmHg, the AUC was 0.669, the sensitivity and specificity to predict the response after POTS intervention (including health education, upright training, ORS, and metoprolol) were 81.8 and 61.7%, respectively. When RPP at HUTT 10 min (RPP10) was 10,988.0 bpm·mmHg, the AUC was 0.769, the sensitivity and specificity were 77.8 and 86.2%, respectively. Liu et al. (71) followed up 57 children with POTS for median of 55 days and found that the reactive group had a longer QTd after intervention (including health education, exercise of autonomic nervous function, ORS, and metoprolol) than the non-responsive group (35 ± 6 vs. 25 ± 5 ms, *p* < 0.001). The AUC was 0.91. Using QTd of 30 ms as a cutoff value, the sensitivity to predict response to POTS intervention is 82.9%, and the specificity is 81.8%. RPP and QTd have prognostic value for POTS, but whether they had prognostic value for specific pharmacological therapy should be further evaluated.

## Conclusion

OI is a clinical syndrome of autonomic regulation disorders. VVS and POTS are more common in school-age children and often occur in early adolescence. Most of the current studies have reported a good overall prognosis for OI, independent of significant mortality. There has been more research on the prognosis of OI in recent years, especially for VVS and POTS, and the predictive value of biomarkers has been gradually popularized in clinical practice. FMD, LVEF and LVFS, BMI, 24-h urinary sodium excretion, MR-proADM, and erythrocyte H_2_S producing rate are relatively stable, non-invasive, and easy to implement biomarkers. Plasma norepinephrine is unstable in blood circulation and the method of detecting CNP has relatively complex operating procedure. The 24-h HRV is affected by physical activity during the day, and hemodynamic change during HUTT is susceptible to emotional effects. However, there is a lack of large, multi-center, and long-term follow-up studies, and the longest follow-up period is about 5.4 years, so the evaluation value of some indicators needs to be further confirmed. If patients have high compliance, early lifestyle change and physical intervention can achieve ideal treatment effects. At the same time, with the help of biomarkers, suitable drugs can be selected for different patients, and even individualized treatment can be realized, which can improve the long-term prognosis of children and adolescents with OI and avoid the occurrence of poor outcomes and even death.

## Summary

For VVS, FMD of 8.85% taken as a cutoff value can be considered as a predictor of the efficacy of MD treatment. LVEF of 70.5% and LVFS of 37.5%, and an increase of 30 beats/min in HR before positive response in HUTT taken as cutoff values can be considered as predictors of the efficacy of metoprolol treatment, respectively. According to the largest AUC (0.906), LVEF was recommended as a predictor of the efficacy of β-blocker therapy on VVS children with priority.

For POTS, when selecting non-pharmacological therapy, QTcd of 43 ms as a cutoff value can be considered as a predictor of the efficacy of physical treatment. Salivary cortisol of 4.1 ng/ml at awakening as a cutoff value can be considered as a predictor of the efficacy of sleep-promoting treatment. A 24-h urine sodium of 124 mmol/24 h, BMI of 18.02 kg/m^2^, MCHC of 347.5 g/L, and HRD between orthostatic and supine position of 41 beats/min combined with HRmax in upright 10 min of 123 beats/min as cutoff values can be considered as predictors of the efficacy of ORS treatment, respectively. For pharmacological therapy, MR-proADM of 61.5 pg/ml, erythrocytic H_2_S of 27.1 nmol/min/10^8^RBC, and FMD of 9.85% as cutoff values can be considered as predictors of the efficacy of MD treatment, respectively. Orthostatic norepinephrine of 3.59 pg/ml, plasma CNP of 32.55 pg/ml, and TR index of 33.7 combined with SDNN index of 79.0 ms, and HR5, HR10, HRD5, HRD10 of 110, 112, 34, 37 beats/min, respectively, as cutoff values can be considered as predictors of the efficacy of metoprolol treatment, respectively. According to the largest AUC (0.923, 0.879, and 0.905, respectively), BMI, MR-proADM, and HRD5 were recommended with priority as predictors of the efficacy of ORS, MD, and metoprolol treatment on POTS in children, respectively.

## Author Contributions

HY conceptualized, prepared, wrote the manuscript, and made the tables. SW, HC, JZ, PL, YW, and RZ participated in providing documentation. RZ and CW reviewed, edited, and revised the manuscript. All authors have read and approved the final manuscript and assume full responsibility for its contents.

## Funding

This work was supported by grants from the Hunan Province Clinical Medical Technology Innovation Guidance Project in China (2020SK53405, 2020SK53406).

## Conflict of Interest

The authors declare that the research was conducted in the absence of any commercial or financial relationships that could be construed as a potential conflict of interest.

## Publisher's Note

All claims expressed in this article are solely those of the authors and do not necessarily represent those of their affiliated organizations, or those of the publisher, the editors and the reviewers. Any product that may be evaluated in this article, or claim that may be made by its manufacturer, is not guaranteed or endorsed by the publisher.
